# Radiation and SN38 treatments modulate the expression of microRNAs, cytokines and chemokines in colon cancer cells in a p53-directed manner

**DOI:** 10.18632/oncotarget.5815

**Published:** 2015-11-05

**Authors:** Surajit Pathak, Wen-Jian Meng, Suman Kumar Nandy, Jie Ping, Atil Bisgin, Linda Helmfors, Patrik Waldmann, Xiao-Feng Sun

**Affiliations:** ^1^ Department of Oncology and Department of Clinical and Experimental Medicine, Linköping University, Linköping, Sweden; ^2^ Department of Gastrointestinal Surgery, West China Hospital, Sichuan University, Chengdu, China; ^3^ Department of Biochemistry & Biophysics, University of Kalyani, Kalyani, West Bengal, India; ^4^ Department of Molecular Biotechnology/IFM, Linköping University, Linköping, Sweden; ^5^ Department of Computer and Information Science, Linköping University, Linköping, Sweden

**Keywords:** colon cancer cells, p53, miRNAs, cytokines, chemokines

## Abstract

Aberrant expression of miRNAs, cytokines and chemokines are involved in pathogenesis of colon cancer. However, the expression of p53 mediated miRNAs, cyto- and chemokines after radiation and SN38 treatment in colon cancer remains elusive. Here, human colon cancer cells, HCT116 with wild-type, heterozygous and a functionally null p53, were treated by radiation and SN38. The expression of 384 miRNAs was determined by using the TaqMan® miRNA array, and the expression of cyto- and chemokines was analyzed by Meso-Scale-Discovery instrument. Up- or down-regulations of miRNAs after radiation and SN38 treatments were largely dependent on p53 status of the cells. Cytokines, IL-6, TNF-α, IL-1β, Il-4, IL-10, VEGF, and chemokines, IL-8, MIP-1α were increased, and IFN-γ expression was decreased after radiation, whereas, IL-6, IFN-γ, TNF-α, IL-1β, Il-4, IL-10, IL-8 were decreased, and VEGF and MIP-1α were increased after SN38 treatment. Bioinformatic analysis pointed out that the highly up-regulated miRNAs, let-7f-5p, miR-455-3p, miR-98, miR-155-5p and the down-regulated miRNAs, miR-1, miR-127-5p, miR-142-5p, miR-202-5p were associated with colon cancer pathways and correlated with cyto- or chemokine expression. These miRNAs have the potential for use in colon cancer therapy as they are related to p53, pro- or anti-inflammatory cyto- or chemokines after the radiation and SN38 treatment.

## INTRODUCTION

Colorectal cancers (CRCs) are the third most common type of cancer, with approximately one million new cases each year world-wide, and the second most frequent cause of cancer-related death in the United States and in Europe [1]. Moreover, it is the second most common site-specific cancer affecting both men and women. Radio- and chemotherapy are the primary treatment in both resectable and advanced CRCs. Radiation improved overall and cancer-specific survival compared to surgery alone [2]. SN38, an active metabolite of irinotecan (or CPT-11), is a water-soluble derivative of camptothecin acting as a topoisomerase I inhibitor. SN38 is thought to exert its antitumor activity *in vivo* after enzymatic cleavage by carboxylesterases 1 and 2 [3]. Although long-standing efforts on early diagnosis and efficient treatment have been made to improve patient survival, but the successes have not been subsequently confirmed, and the benefits of radio- and chemotherapy are still under investigation. The identification of molecular biomarkers and other therapeutic target has been the focus of extensive research where the ultimate goal is to discover markers with a diagnostic and/or therapeutic value. In most cases, it is not clear what causes colon cancer, although several risk factors have been identified over the years. Recently, inflammation in the colon has been implicated in development of colon cancer and its role has been validated by many excellent epidemiological and experimental studies [4, 5]. Activated inflammatory cells produce reactive oxygen species (ROS) and reactive nitrogen intermediates that can induce DNA damage and mutation [6]. However, in response to DNA damage, it is also well established that p53 is an important factor, whereas, p53-mutant cells are resistant to drug-induced apoptosis [7]. It has been shown that colon cancer cells are sensitive to different treatments depending on p53 status [8]. On the contrary, cyto- and chemokines can serve as tumor growth and survival factors, and can promote or reduce tumor growth [9]. Commonly, after a tumor forms, the localized inflammatory microenvironment can promote the accumulation of additional mutations and epigenetic changes. One of the epigenetic regulator, microRNAs (miRNAs), a small non-coding RNAs of 18–24 nucleotides regulates gene expression by translational repression or cleavage of the mRNA targets [10]. miRNAs are involved in various biological processes including cell proliferation, differentiation and apoptosis [11]. Expression of many miRNAs is up- or down-regulated in tumors compared to normal tissues, including CRCs [12]. Further, a large number of evidence suggests that miRNAs is involved in modulating the chemosensitivity and chemoresistance of tumor cells [13]. Each miRNA has the ability to control the activity of hundreds of target genes, including oncogenes and tumor suppressors, like p53 [14], although miRNA expression in relation to radiation, SN38, and increase or decrease of cyto- or chemokine expression is less investigated.

The present study aimed to investigate the p53 gene mediated expression of miRNAs, cyto- and chemokines in human colon cancer cells (HCT116) after the treatments of radiation and SN38, and further examined the most significantly up- or down-regulated miRNAs to find out whether there is any possible interaction between these miRNAs and increased or decreased cyto- and chemokine expression in colon cancer cells in response to the radiation and SN38 treatments. This study is hypothesized to find out a possible link between the expression of miRNAs, cyto-, chemokines and p53 gene after the treatment of radiation and SN38 in colon cancer cells; this might predict miRNAs, as a therapeutic target in future colon cancer therapy.

## RESULTS

### ID_50_ of radiation and IC_50_ of SN38 treatments in HCT116 cells

Increasing dose (2Gy-10Gy) of radiation was used to test the cellular viability of HCT116^p53+/+^, HCT116^p53+/−^ and HCT116^p53−/−^, and 2Gy radiation is used as an ID_50_ value for all the subsequent experiments. Cellular viability of the three cell lines after exposure to increasing concentrations of SN38 (0–1 μM) was examined by using the XTT assay. The IC_50_ value for HCT116^p53+/+^ cells was found to be 100 nM, 150 nM for HCT116^p53+/−^ and 300 nM for HCT116^p53−/−^cells, respectively. IC_50_ is the maximal concentration of SN38 to cause 50% inhibition of biological activity of the cells. Similarly ID_50_ is the maximal radiation dose to cause the half of the inhibition of the cells. Percentage of inhibition of cells is different for different cell types, and percentage of inhibition depends on the responses of the particular cancer cell types to particular radiation and drug. The IC_50_ and ID_50_ dosages were used for all further experiments.

### Expression of miRNAs in HCT116 cells after radiation and SN38 treatment

**Figure 1:**
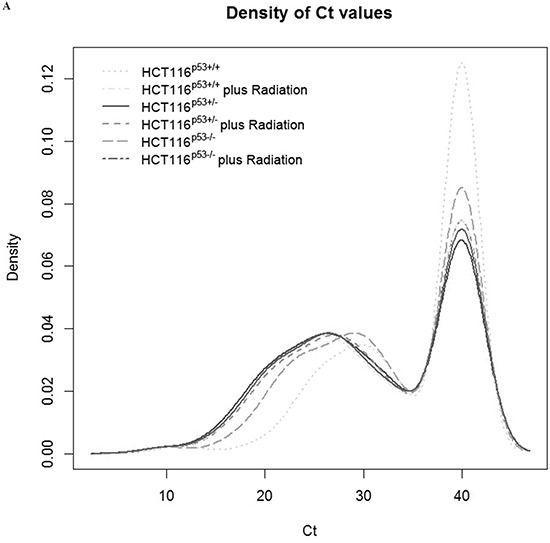

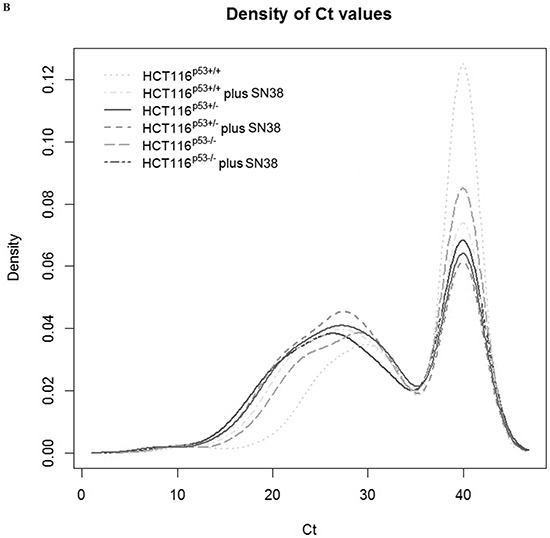
Distribution of the density of raw Ct values for the individual samples without normalization The expression levels of 379 miRNAs and 5 control assays were analyzed in HCT116^p53+/+^, HCT116^p53+/−^ and HCT116^p53−/−^. **A.** is for the expression of miRNA without or with the radiation treatment. **B.** is for the expression of miRNA without or with the SN38 treatment. The both A and B illustrate that the different cell lines had almost the same trends of raw Ct values.

Expression levels of 379 miRNAs and 5 control assays were analyzed in HCT116^p53+/+^, HCT116^p53+/−^ and HCT116^p53−/−^ after radiation and SN38 treatment. Figure 1 illustrates that the different cell lines had almost the same trends of raw Ct values. The data were presented as log_2_ (T/N) values where T was considered as treatment and N was as untreated. The levels of miRNA expression were changed by the treatments, and the results depended on p53 gene status in the different cell lines. Log_2_ (T/N) value > 0.5 of miRNA expression was considered as up-regulation

**Figure 2:**
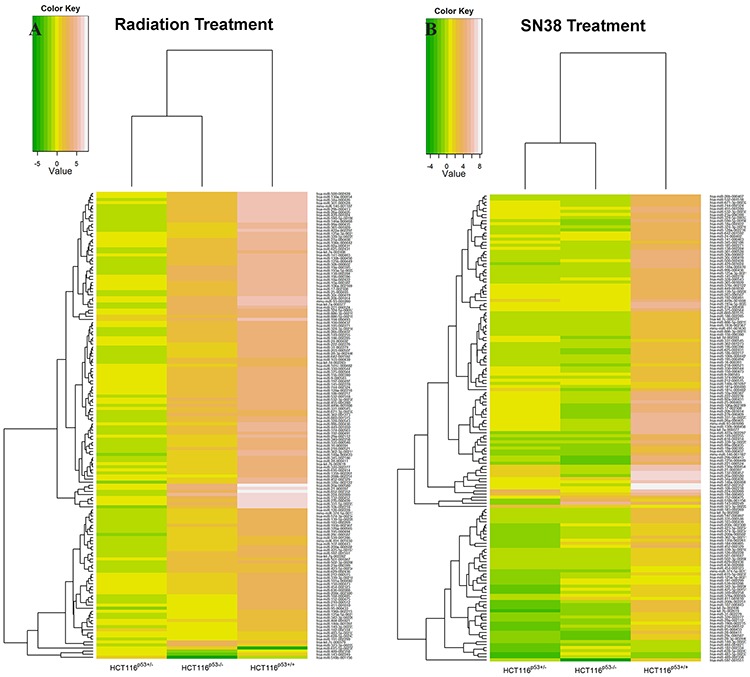
Heatmaps for the expression of miRNAs in HCT116^p53+/+^, HCT116^p53+/−^ and HCT116^p53−/−^ cell lines with the radiation and SN38 treatment, the cell lines grouped in vertical columns and miRNAs arranged horizontally by the similarity of the expression to one another The clusters were based on the Euclidean distance between log_2_ (T/N) values. T/N= Treatment/Normal, and > 0.5 of miRNA expression was considered as up-regulation. **A.** is for the expression of miRNA after the radiation treatment, and **B.** is for the expression of miRNAs after SN38 treatment. The more white color shows the more up-regulation, and the more green color is for the more down-regulation. Here only those miRNAs with log_2_ (T/N) values from all the three cell lines were considered in the heatmap. HCT116^p53+/+^ cells had more up-regulated miRNAs and HCT116^p53−/−^ cells had more down-regulated miRNAs of the each treatment.

Figure 2 displays an overview of human miRNA expression after radiation (A) and SN38 (B) treatments in a euclidean based heatmap, along with a dendrogram, with the cell lines grouped in vertical columns and miRNAs arranged horizontally by the similarity of the expression to one another. We focused our attention on these confidently detected miRNAs so that the downstream analysis was based on the most reliable expression data. miRNAs that were part of the same family showed highly correlated expression. The HCT116^p53+/+^ cells had most up-regulated miRNAs while HCT116^p53−/−^ cells had most down-regulated ones in the both radiation and SN38 treatments, although miRNAs were expressed in a much higher level under radiation treatment compared to SN38 treatment. In the both treatments, miRNAs were divided into two major groups, and each of the group was divided into the number of sub-groups in a similar fashion depending on their expression pattern.

**Figure 3:**
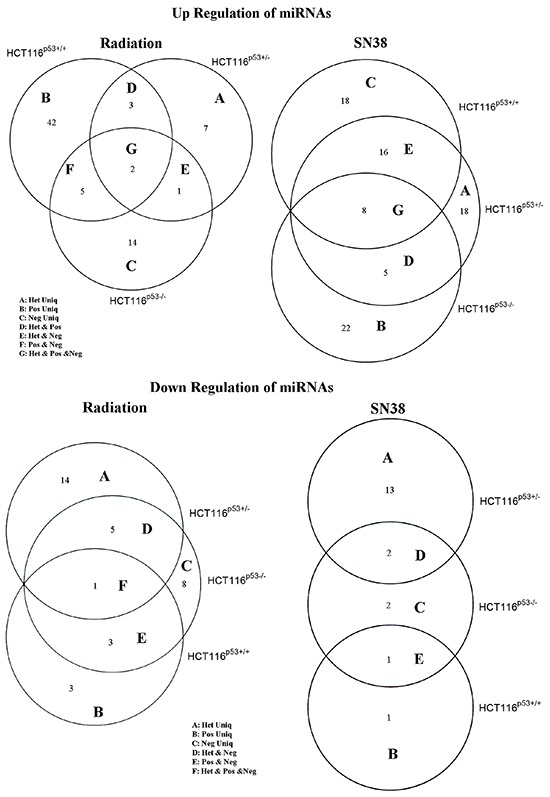
Venn diagram showing up-regulated A. and down-regulated B. miRNAs after radiation and SN38 treatment in HCT116^p53+/+^, HCT116^p53+/−^ and HCT116^p53−/−^ cell lines The Empirical Bayes mixture method implemented in the R-package EBarrays was used for analyzing differential expression (DE) of miRNAs in the cells. The numbers of overlapping significant miRNAs between the cell lines were visualized with Venn-diagrams.

To study the p53 gene dependent or independent expression of miRNAs after the radiation or SN38 treatment in the cells, we used Empirical Bayes method (EBarrays), and the results are presented in Figure 3A and 3B and Table 1a–1d1. Irrespective of p53 status, after radiation miR-302a and miR-302c up-regulated, and miR-518f down-regulated in the all cell lines, whereas after SN38 treatment up-regulated miRNAs were miR-133a, miR-155-3p, miR-204, miR-22, miR-512-3p, miR-517a, miR-517c and miR-708 in the all cell lines (Figure 3, Table 1a).

**Table 1a:** Up-regulated miRNAs in HCT116^p53+/+^, HCT116^p53+/−^ and HCT116^p53−/−^ cells after radiation and SN38 treatment

Treatment	miRNA	HCT116^p53+/+^	HCT116^p53+/−^	HCT116^p53−/−^
**Radiation**	miR-302a	4.655981439	0.86155842	7.861353702
	miR-302c	7.773350237	1.276794405	10.66082578
**SN38**	miR-133a	7.8622282	4.392523507	8.462469553
	miR-155-3p	8.206027775	10.8408031	7.841954255
	miR-204	7.197914523	10.56998658	3.827717423
	miR-22	11.4619751	13.92959407	11.27147879
	miR-512-3p	9.679172174	5.89866789	9.042581415
	miR-517a	7.378598321	10.85739323	7.154980087
	miR-517c	7.680176647	11.25826628	7.149330507
	miR-708	7.948256251	2.755742179	4.892050957

T/N= Treatment/Normal, > 0.5 =up-regulation, Data was presented as Log2 (T/N +/+)

In HCT116^p53+/+^ cells, after radiation 42 miRNAs were up-regulated (let-7f, miR-101, miR-135a, miR-139-3p, miR-142-3p, miR-148b, miR-190, miR-193a-3p, miR-199a-3p, miR-202-3p, miR-211, miR-219-1-3p, miR-223, miR-296, miR-301b, miR-32, miR-33b, miR-361, miR-372, miR-376b, miR-410, miR-424, miR-433, miR-450a, miR-455-3p, miR-487b, miR-502, miR-505, miR-511, miR-545, miR-548b-5p, miR-548c, miR-548d, miR-548d-5p, miR-561, miR-570, miR-576-3p, miR-576-5p, miR-652, miR-655, miR-885-5p, miR-98), and after the SN38 treatment 22 miRNAs were up-regulated (let-7f, miR-101, miR-135a, miR-139-3p, miR-142-3p, miR-148b, miR-199a-3p, miR-205, miR-219-1-3p, miR-223, miR-296, miR-301b, miR-302b, miR-32, miR-361, miR-372, miR-455-3p, miR-502, miR-505, miR-545, miR-576-3p, miR-627), whereas after the radiation only 3 miRNAs (miR-1, miR-510, miR-615-5p) and one after SN38 (miR-510) were down-regulated (Figure 3, Table 1b1 and Table 1b2).

**Table 1b1:** Up- and down-regulated miRNAs in HCT116^p53+/+^ cells after radiation treatment

Regulation	miRNA	HCT116^p53+/+^
**Up**	let-7f	12.10081869
	miR-101	10.49355727
	miR-135a	8.995148115
	miR-139-3p	11.07415016
	miR-142-3p	10.11289948
	miR-148b	11.70230864
	miR-190	5.020591095
	miR-193a-3p	6.938944406
	miR-199a-3p	10.33499166
	miR-202-3p	5.901977075
	miR-211	9.170616127
	miR-219-1-3p	10.40630388
	miR-223	8.586243075
	miR-296	7.053426348
	miR-301b	11.81352228
	miR-32	10.30348624
	miR-33b	5.564743979
	miR-361	11.17827581
	miR-372	9.365062585
	miR-376b	4.436961338
	miR-410	6.788829242
	miR-424	6.319509555
	miR-433	4.8717451
	miR-450a	6.778011402
	miR-455-3p	11.73009094
	miR-487b	8.397537736
	miR-502	9.031097398
	miR-505	8.20027089
	miR-511	5.178674809
	miR-545	9.801365628
	miR-548b-5p	6.233542987
	miR-548c	4.615828235
	miR-548d	8.13769307
	miR-548d-5p	7.750298318
	miR-561	5.237678432
	miR-570	5.436495018
	miR-576-3p	10.61378486
	miR-576-5p	6.248838402
	miR-652	7.989939399
	miR-655	5.095797862
	miR-885-5p	8.412637587
	miR-98	11.87928381
**Down**	miR-1	−7.965784285
	miR-615-5p	−4.717856771
	miR-510	Undetected

**Table 1b2:** Up- and down-regulated miRNAs in HCT116^p53+/+^ cells after SN38 treatment

Regulation	miRNA	HCT116^p53+/+^
**Up**	let-7f	12.36350238
	miR-101	9.667608326
	miR-135a	9.201565253
	miR-139-3p	11.1732655
	miR-142-3p	8.863284953
	miR-148b	10.88900574
	miR-199a-3p	8.86613216
	miR-205	8.231586009
	miR-219-1-3p	9.770407868
	miR-223	8.363622287
	miR-296	7.266111876
	miR-301b	11.66905601
	miR-302b	7.832782441
	miR-32	8.698892153
	miR-361	10.74891318
	miR-372	9.66178166
	miR-455-3p	12.24254803
	miR-502	7.333405843
	miR-505	8.587773767
	miR-545	7.859149602
	miR-576-3p	10.15602491
	miR-627	9.164298554
**Down**	miR-510	Undetected

T/N= Treatment/Normal, > 0.5 =up-regulation, Data was presented as Log2 (T/N +/+).

In HCT116^p53+/−^ cells, after the radiation 7 miRNAs were up-regulated (miR-199b, miR-342-5p, miR-518b, miR-519d, miR-520b, miR-556-3p, miR-654-3p), and after SN38 treatment 18 miRNAs were upregulated (miR-1, miR-122, miR-133b, miR-143, miR-193a-3p, miR-202-3p, miR-214, miR-34c, miR-370, miR-382, miR-450a, miR-494, miR-506, miR-518b, miR-519e, miR-523, miR-598, miR-891a) (Figure 3, Table 1c1 and Table 1c2). Whereas after radiation 14 miRNAs were down-regulated (miR-127-5p, miR-133a, miR-142-5p, miR-215, miR-219, miR-326, miR-371-3p, miR-486, miR-508, miR-518d, miR-520d-5p, miR-548a-5p, miR-589, miR-708), and after SN38 treatment 13 miRNAs were down-regulated (miR-142-5p, miR-146b-3p, miR-190, miR-215, miR-219, miR-326, miR-518d, miR-520d-5p, miR-548a-5p, miR-548d, miR-597, miR-655, miR-889).

**Table 1c1:** Up- and down-regulated miRNAs in HCT116^p53+/−^ cells after radiation treatment

Regulation	miRNA	HCT116^p53+/−^
**UP**	miR-199b	7.718245165
	miR-342-5p	5.730830129
	miR-518b	0.860764203
	miR-519d	3.484138131
	miR-520b	0.621055503
	miR-556-3p	5.288801492
	miR-654-3p	7.046578367
**Down**	miR-127	−8.965784285
	miR-133a	−6.265344567
	miR-142-5p	−7.158429363
	miR-215	−9.965784285
	miR-219	−4.506352666
	miR-326	−3.836501268
	miR-371-3p	−7.64385619
	miR-486	−7.158429363
	miR-508	−7.158429363
	miR-518d	−4.411195433
	miR-520d-5p	−4.13289427
	miR-548a-5p	−5.380821784
	miR-589	−2.795859283
	miR-708	−5.717856771
	miR-142-5p	−7.158429363

**Table 1c2:** Up- and down-regulated miRNAs in HCT116^p53+/−^ cells after SN38 treatment

Regulation	miRNA	HCT116^p53+/−^
**UP**	miR-1	0.655351829
	miR-122	7.161535025
	miR-133b	7.205412064
	miR-143	5.02467428
	miR-193a-3p	3.792022388
	miR-202-3p	7.740570079
	miR-214	7.188994276
	miR-34c	9.931790889
	miR-370	7.68648652
	miR-382	1.21556091
	miR-450a	7.979247974
	miR-494	2.36232961
	miR-506	8.360719814
	miR-518b	3.534310665
	miR-519e	3.49646235
	miR-523	7.809401581
	miR-598	7.238538493
	miR-891a	6.436628267
**Down**	miR-146b-3p	−6.158429363
	miR-190	−7.158429363
	miR-215	−9.965784285
	miR-219	−4.380821784
	miR-326	−3.736965594
	miR-518d	−4.321928095
	miR-520d-5p	−4.011587974
	miR-548a-5p	−5.265344567
	miR-548d	−2.775959726
	miR-597	−4.965784285
	miR-655	−2.826232932
	miR-889	−2.158429363

In HCT116^p53−/−^ cells, after the radiation 14 miRNAs were up-regulated (miR-127, miR-133a, miR-142-5p, miR-155-5p, miR-224, miR-329, miR-330-5p, miR-367, miR-485-3p, miR-522, miR-589, miR-708, miR-888, miR-889), and after SN38 treatment 18 miRNAs were up-regulated (miR-127, miR-142-5p, miR-219, miR-302a, miR-302c, miR-330-5p, miR-371-3p, miR-380-3p, miR-450b-5p, miR-485-3p, miR-487a, miR-518d, miR-548a, miR-548a-5p, miR-548b-5p, miR-561, miR-589, miR-618) and 8 miRNAs (miR-211, miR-410, miR-433, miR-450a, miR-491-3p, miR-494, miR-520b, miR-672) and 2 miRNAs (miR-202-5p, miR-433) were down-regulated, respectively (Figure 3, Table 1d1 and Table 1d2). Our result showed that up- or down-regulations of miRNAs in the colon cancer cells largely depended on the type of the treatments and p53 status in colon cancer cells.

**Table 1d1:** Up- and down-regulated miRNAs in HCT116^p53−/−^ cells after radiation treatment

Regulation	miRNA	HCT116^p53−/−^
**Up**	miR-133a	5.177000871
	miR-142-5p	1.862724134
	miR-155-5p	11.91291627
	miR-224	4.499463243
	miR-329	5.827488793
	miR-330-5p	7.79881522
	miR-367	5.793427737
	miR-485-3p	8.705836251
	miR-522	5.671095186
	miR-589	6.06063109
	miR-708	4.23319783
	miR-888	6.656625075
	miR-889	7.277631467
	miR-127	1.404758396
**Down**	miR-211	−1.49410907
	miR-410	−0.60823228
	miR-433	−7.380821784
	miR-450a	−8.380821784
	miR-491-3p	−1.275786313
	miR-494	−2.300448367
	miR-520b	−4.035046947
	miR-672	−3.943416472

**Table 1d2:** Up- and down-regulated miRNAs in HCT116^p53−/−^ cells after SN38 treatment

Regulation	miRNA	HCT116^p53−/−^
**UP**	miR-142-5p	5.128747059
	miR-219	3.56315813
	miR-302a	6.411985453
	miR-302c	6.991748578
	miR-330-5p	6.753845179
	miR-371-3p	6.212588793
	miR-380-3p	6.03771216
	miR-450b-5p	7.443482333
	miR-485-3p	7.059571596
	miR-487a	4.603656015
	miR-518d	3.788372501
	miR-548a	6.111156541
	miR-548a-5p	3.163820726
	miR-548b-5p	2.89947512
	miR-561	5.671859468
	miR-589	4.174406419
	miR-618	3.808282071
**Down**	miR-433	−8.965784285
	miR-202-5p	−9.965784285

T/N= Treatment/Normal, > 0.5 =up-regulation, Data was presented as Log2 (T/N −/−)

### Pathway of miRNAs

Based on our experimental results and EBarrays statistical analysis, let-7f-5p and miR-455-3p were up-regulated more than 12 times after radiation and SN38 treatment in HCT116^p53+/+^ cells compared to untreated cells, whereas they were down-regulated in HCT116^p53+/−^ cells and marginally increased (>3 times) in HCT116^p53−/−^ cells. The same trend was found in case of miR-98, whereas miR-155-5p was up-regulated mostly (>12 times) in HCT116^p53−/−^ cells after radiation and in HCT116^p53+/+^ cells (>7 times) after SN38 treatment. miR-1 was down-regulated after radiation and SN38 treatment in HCT116^p53+/+^ and HCT116^p53−/−^ cells most significantly (undetected) but after SN38 treatment in HCT116^p53+/−^ cells it was marginally up-regulated. In case of miR-127-5p and miR-142-5p the most notable down-regulation was found in HCT116^p53+/+^ and HCT116^p53+/−^ cells after the treatment, whereas miR-127-5p and miR-142-5p were up-regulated in HCT116^p53−/−^ cells after the radiation treatment. On the other hand, miR-202-5p was down-regulated (>9.5 times) in HCT116^p53−/−^ cells after the treatment. We found these four up-regulated and four down-regulated miRNAs most significantly changed their expression pattern (in terms of fold change) after the treatments compared to other miRNAs analyzed. The changes in the expression of these miRNAs were also largely depending on the p53 status in colon cancer cells. To validate our results found in HCT116 cells, we further examined the expression of miRNAs, let-7f-5p, miR-455-3p, miR-98, miR-155-5p, miR-1, miR-127-5p, miR-142-5p and miR-202-5p in KM12C and KM12L4a human colon cancer cell lines. We used 2Gy radiation and 150 nM SN38 for the subsequent experiments. The results showed that let-7f-5p, miR-455-3p, miR-98 and miR-155-5p were up-regulated in KM12C (Supplementary Figure S1) and KM12L4a (Supplementary Figure S2) cell lines after radiation and SN38 treatment, whereas miR-1, miR-127-5p, miR-142-5p and miR-202-5p were undetected in KM12C and KM12L4a cell lines after radiation and SN38 treatment.

**Figure 4:**
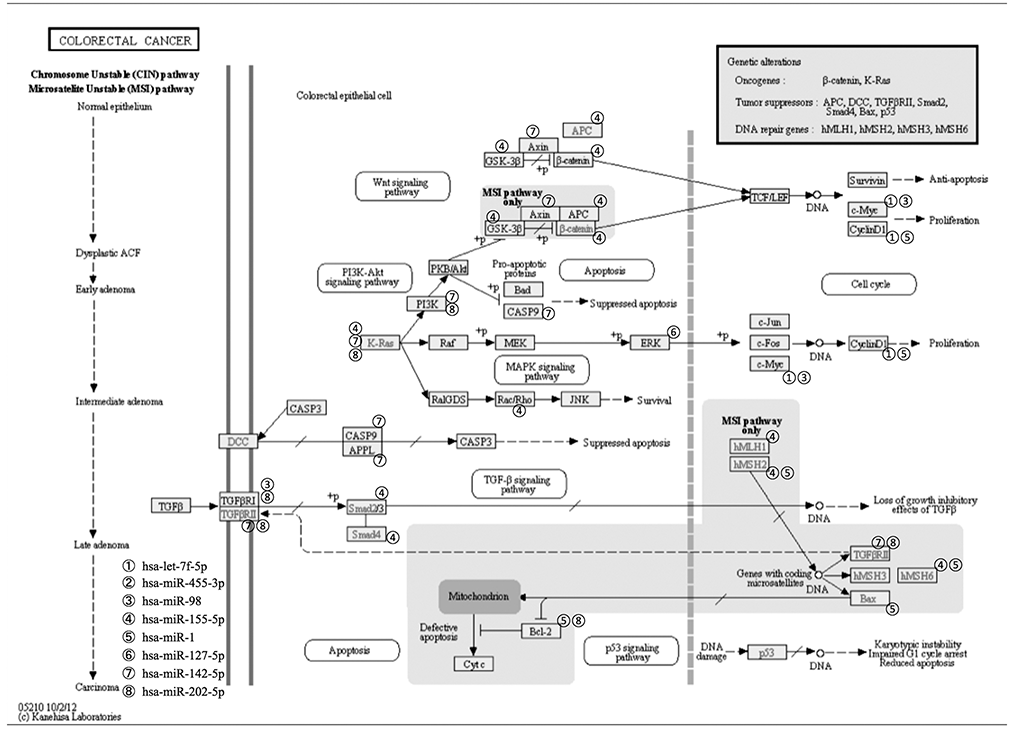
Colorectal cancer pathway with interaction between significant up-regulated miRNAs, let-7f-5p, miR-455-3p, miR-98, miR-155-5p, and down-regulated miRNAs, miR-1, miR-127-5p, miR-142-5p, miR-202-5p after radiation and SN38 treatments and their target genes

**Figure 5:**
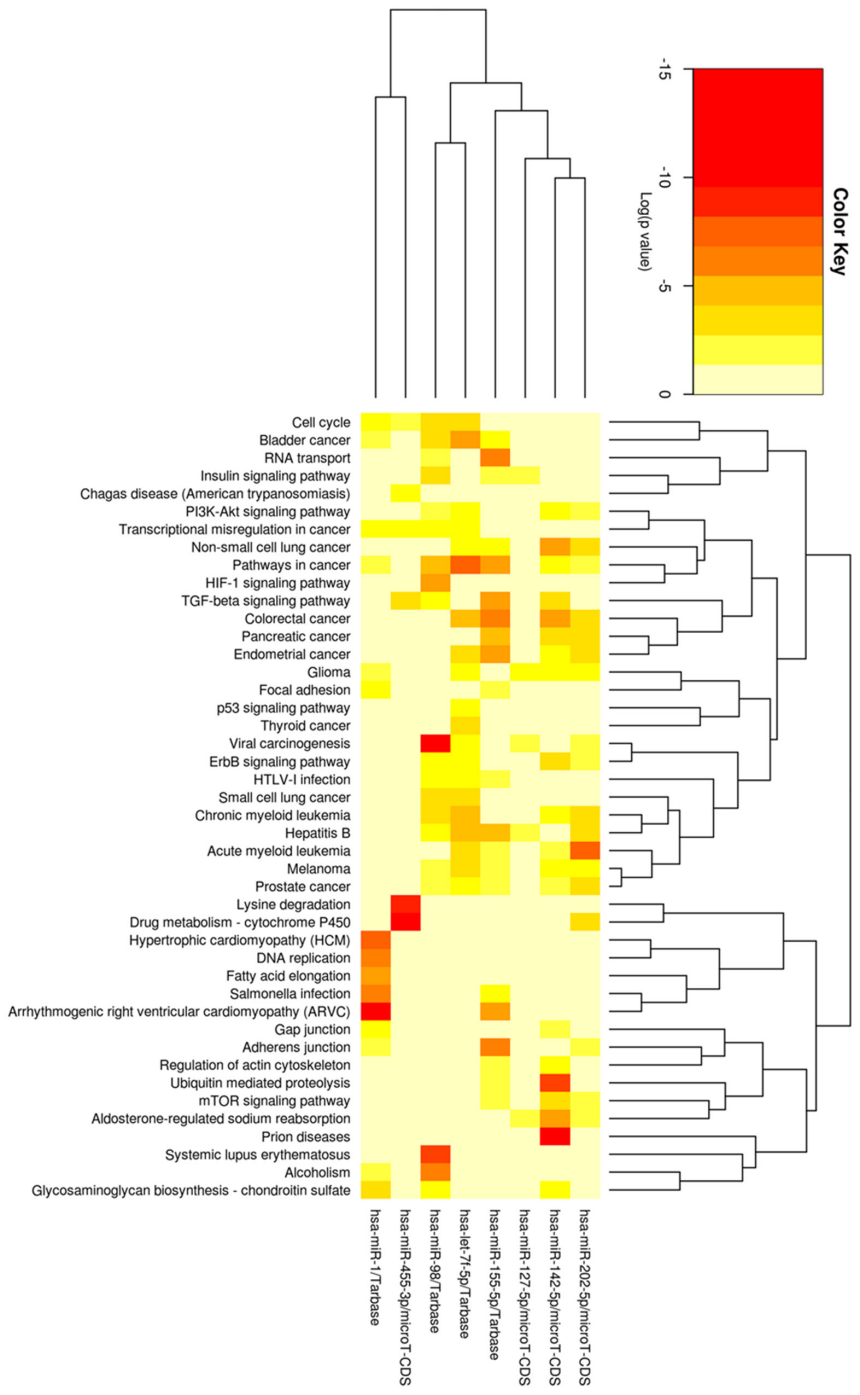
Heatmap of four most significant up-regulated miRNA, let-7f-5p, miR-455-3p, miR-98 and miR-155-5p, as well as four most down-regulated miRNA, miR-1, miR-127-5p, miR-142-5p and miR-202-5p after radiation and SN38 treatments, and different pathways by clustering from pathway union Each colorful square means that pathway contains target genes of the miRNA. The more red color shows the more significant relationship between the miRNA and the pathway.

So, we were interested to further study the pathways of these miRNAs and to predict their relationships with KEGG pathways by using DIANA-miRPath tool. Figure 4 indicates their relationships with the pathway of colon cancer and almost every sub-pathway contains target genes of the 8 miRNAs. Target genes of the 8 miRNAs are distributed in all the sub-pathways, especially PI3K-Akt signaling pathway and Wnt signaling pathway. K-ras is the mostly target gene of these miRNAs. These two pathways along with K-ras pathway have been proven by previous studies to play key roles in the overall colorectal cancer pathway. Figure 5 shows the heatmap of these miRNAs and different pathways by clustering from pathway union.

### Cyto- and chemokine expression in HCT116 cells after radiation and SN38 treatment

**Figure 6:**
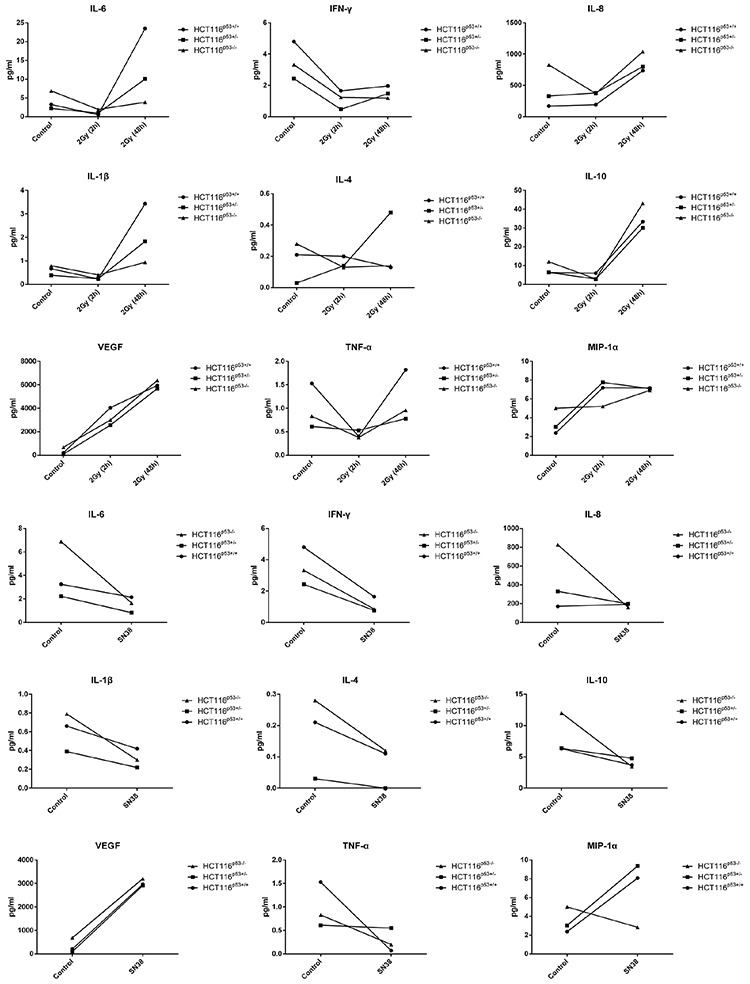
The expression of cytokine and chemokine in HCT116^p53+/+^, HCT116^p53+/−^ and HCT116^p53−/−^ cell lines after radiation A. and SN38 treatments B Cytokines, IL-6, TNF-α, IL-1β, Il-4, IL-10, VEGF, chemokines, IL-8, MIP-1α were increased, and IFN-γ expression was decreased after 48 hr of 2GY radiation, whereas, IL-6, IFN-γ, TNF-α, IL-1β, Il-4, IL-10, IL-8 expression were decreased, and VEGF MIP-1α were increased after SN38 treatment. For all of the experimental samples and all of the cyto- and chemokines a z-score was calculated as $z=\frac{X-\mu }{\sigma }$. If z-score was greater than 1.96 then it up-regulated and down-regulated if the z-score was less than 1.96, corresponding to a *p*-value of 0.05.

Out of the 20 cytokines [GM-CSF, IL-15, IL-1α, IL-16, IL-5, IL-17, IL-7, TNF-β, IL-12p40, VEGF, IFN-γ, IL-8 (high-ab), IL-1β, IL-10, IL-2, IL-12p70, IL-4, IL-13, IL-6, TNF-α] and 10 chemokines [MIP-1α, MIP-1β, TARC, Eotaxin, Eotaxin-3, MCP-1, MCP-4, MDC, IP-10, IL-8 (chem)], noticeable increase or decrease was found in the expression of cytokines, IL-6, TNF-α, IFN-γ, IL-1β, Il-4, IL-10, VEGF, and of chemokines, the IL-8 and MIP-1α after 2GY radiation (Figure 6A) and SN38 (Figure 6B). After 2Gy radiation treatment, TNF-α and IFN-γ expression were seen to be reduced in three cell lines, HCT116^p53+/+^, HCT116^p53+/−^ HCT116^p53−/−^, but the reduction of TNF-α was pronounced in HCT116^p53+/+^ cells as compared to the other two cell lines. Furthermore, we observed increased expression of MIP-1α and VEGF in all the cell lines treated under the similar radiation condition compared to unradiated cells. Moreover, after 48 hr of 2Gy radiation exposure, significant increase of IL6, IL-1β, IL-4, IL-10, VEGF, TNF-α cytokines as well as IL-8 and MIP-1α chemokine expression were noticed in all cell lines compared to unradiated cells (Figure 6A). Reduced IL-6 expression was observed after the SN38 treatment in the all cell lines irrespective with their p53 status. A similar trend of reduction was noticed in case of IFN-γ, IL-1β, IL-4, and even in IL-10 expression. Furthermore, TNF- α expression was seen to be reduced in HCT116^p53+/+^ and HCT116^p53−/−^ cells but in HCT116^p53+/−^ cells no noticeable changes was found when compared with untreated cells (Figure 6B). Increased MIP-1α and VEGF were observed after SN38 treatment in HCT116^p53+/+^ and HCT116^p53+/−^ but MIP-1α in HCT116^p53−/−^ cells after the SN38 treatment was reduced (Figure 6B).

### *In silico* interaction of deregulated miRNAs and cyto- and chemokine expression in HCT116 cells

**let-7f-5p**: RNA22 predicts interaction between let-7f-5p and interleukin 4. According to TargetScan, PicTar, miRanda, DIANA-microT let-7f-5p can also directly interact with IL-6. PicTar tells about possible let-7f-5p, PicTar, miRanda, DIANA-microT and RNA22 again report multiple interaction sites for let-7f-5p in IL-10. TargetScan and miRanda both find the same interaction site of let-7f-5p in MIP-1αand IL-8 chemokine interaction, while DIANA-microT reports another. RNA22 identifies three possible sites of interaction between VEGF and let-7f-5p. **miR-98**: TargetScan, PicTar, miRanda, DIANA-microT altogether predicts its interaction with IL-6. TargetScan, PicTar and DIANA-microT show interactions with IL-10 at the same site. PicTar reports possible interactions of has-miR-98 with IL-8. **miR-455-3p**: Direct interaction of miR-455-3p with IL-1β is predicted by DIANA-microT, PITA and RNA22 servers. DIANA-microT and RNA22 v2 both recognize the same site for hsp-miR-98 and chemokine MIP-1- α interaction. RNA22 also speculates its interaction with VEGF. **miR-155-5p**: RNA22 detects interaction between miR-155-5p and interleukin 1βand miRanda, RNA22 both spot interaction site in VEGF for miR-155-5p. **miR-1**: An interaction site of miR-1 is reported in IL-10 by DIANA-microT, while TargetScan, PicTar, RNA22, miRanda, and DIANA-microT recognize multiple probable interactions between VEGF and has-miR-1. **miR-127-5p**: RNA 22 predicts multiple site interaction between miR-127-5p and IL-1beta. RNA22 and DIANA-microT describe possible interactions with VEGF, while RNA22 alone foretells about its interaction with TNF- α. **miR-142-5p**: miRanda points towards possible interactions of has-miR-142-5p with IL-6. **miR-202-5p**: TargetScan, PicTar, and miRanda foretell about miR-202-5p interaction with IL-6. RepTar describes two interaction sites of has-miR-202 in IL-10. RNA22 detects probable miR-202-5p interactions with chemokine MIP-1 α. PicTar also forecasts interaction with IL-8.

## DISCUSSION

The mechanisms underlying colon cancer remain subjects of extensive investigation in the cancer biology field. It is known that colon cancer results from accumulation of both genetic and epigenetic alterations of the cellular genome, which transforms normal glandular epithelium into adenocarcinoma [15]. Increased or decreased expression of miRNAs, cyto- and chemokines are associated with development and progression of various cancers by stimulating the translation of oncogenes and tumor suppressor genes. Such as, the tumor suppressor gene p53 is mutated in about 70% of CRCs and it is an essential step in the development of CRCs [16]. The mechanism by which p53 is inactivated in the evolution of cancer can have important consequences on responsiveness to cancer treatment. For example, p53 mutation renders HCT116 colon cancer cells more sensitive to radiation and adriamycin but less sensitive to 5-fluorouracil [17]. A further complication is that p53 appears to respond to RNA damage rather than DNA damage in response to 5-fluorouracil treatment [17]. The interaction of p53, miRNAs and cytokines as prognostic classifiers and their functional role in the development of metastases were studied in different types of cancers including colon cancer [18–22]. Generally, epigenetic alterations not only complement genetic alterations in the colon cancer, but also complex interactions between different DNA, alteration of cytokine production and chromatin modifications exist which characterize subgroups of colon cancer with distinct etiology and prognosis. Although, long-standing efforts on early diagnosis and efficient treatment have been made to improve patient survival, the successes have not been subsequently confirmed, and the benefits of radio-, and/or chemotherapy are still under investigation [23]. In the present study, we examined the expression of miRNAs in HCT116^p53+/+^, HCT116^p53+/−^ and HCT116^p53−/−^ cell lines after radiation and SN38 treatment. Furthermore, we used Empirical Bayes method (EBarrays) to study differentially expressed miRNAs between untreated and treated cells based on their p53 status. Wild type (wt) p53 (+/+) can induce apoptosis, cell cycle arrest and senescence, which are sufficient to explain tumor suppression by p53 [24]. Irrespective of wild type or mutated or null p53, after radiation treatment, miR-302a and miR-302c up-regulated, and miR-518f down-regulated in colon cancer cells, whereas after SN38 treatment up-regulated miRNAs were miR-133a, miR-155, miR-204, miR-22, miR-512-3p, miR-517a, miR-517c and miR-708 in the all colon cancer cell lines. Most of these miRNAs are not extensively studied till now. However, the majority of the miRNAs were dys-regulated by radiation and SN38 treatment was largely dependent on p53 status of the HCT116 cells, i.e., either wt type or mutated p53. Generally, p53 suppresses the conversion from arrest to senescence (geroconversion) [25], Wt p53 seems to have three independent effects: apoptosis, cell-cycle arrest and gerosuppression. By inducing arrest, wt p53 primes cells for senescence, Mice with increased, but normally regulated, p53 lives longer [26]. p53 knockout mice have both accelerated carcinogenesis and decreased longevity [27]. While the loss of p53 by itself makes cells prone to become tumorigenic, an increased rate of organismal aging in the absence of p53 may further accelerate carcinogenesis.

In HCT116^p53+/+^ cells, after the radiation 42 miRNAs and after SN38 treatment 22 miRNAs were up-regulated, whereas after the radiation only 3 miRNAs and one after SN38 were down-regulated. In HCT116^p53+/−^ cells, after the radiation 7 miRNAs were up-regulated and after SN38 treatment 18 miRNAs were up-regulated, whereas after radiation 14 miRNAs and after SN38 treatment 13 miRNAs were down-regulated. In HCT116^p53−/−^ cells, after the radiation 14 miRNAs were up-regulated and after SN38 treatment 18 miRNAs were up-regulated and 2 miRNAs were down-regulated, respectively. Moreover, from EBarrays, we found miRNAs, such as let-7f-5p, miR-455-3p, miR-98, miR-155-5p, up-regulated to the highest degree and miRNAs, miR-1, miR-127-5p, miR-142-5p, miR-202-5p were down-regulated most, after the radiation and SN38 treatment. Changes in the expressions of these miRNAs were also largely depending on p53 status. Such as, let-7f-5p and miR-455-3p and miR-98 were up-regulated most after radiation and SN38 treatment in HCT116^p53+/+^ cells, whereas miR-155-5p was up-regulated mostly in HCT116^p53−/−^ cells after radiation and in HCT116^p53+/+^ cells after SN38 treatment. mir-1 was down-regulated after the radiation and SN38 treatment in HCT116^p53+/−^ and HCT116^p53−/−^ cells. In miR-127-5p and miR-142-5p the highest degree of down-regulation was found in HCT116^p53+/+^ and HCT116^p53+/−^ cells after the radiation treatment. miR-202-5p was down-regulated in HCT116^p53−/−^ cells after the SN38 treatment. We also found in KM12C and KM12L4a cells with p53 mutation, let-7f-5p, miR-455-3p, miR-98, miR-155-5p and miRNAs were up-regulated, whereas miR-1, miR-127-5p, miR-142-5p and miR-202-5p remained undetected after the radiation and SN38 treatment.

We then were interested to study the pathways of these miRNAs and to predict their relationships with KEGG pathways by using DIANA-miRPath tool. Relationships with the pathway of CRCs and almost every sub-pathway contain target genes of these 8 miRNAs as shown in Figure 4. Target genes of the 8 miRNAs are distributed in all the sub-pathways, especially PI3K-Akt signaling pathway and Wnt signaling pathway, although K-ras is the mostly target gene of these miRNAs. These pathways have been proven to play key roles in the overall pathway of CRC development. Moreover, Let-7 mi-RNA precursor (includes let-7f-5p and miR-98) has been demonstrated to be a direct regulator of *RAS* expression in human cells [28], and direct inhibitor of *HMGA2* by binding to its 3′UTR [29]. Expression level of *let-7* is already proposed as a prognostic marker in several types of cancers [30]. Increased expression of let-7 miRNA is also associated with CRC [31]. p53 plays a key role in induction of both tristetraprolin and let-7 in human cancer cells [32]. Intranasal administration of let-7 has already been found effective in reducing tumor growth in a transgenic mouse model of lung cancer and similar restoration of let-7 was also shown to inhibit cell proliferation in several types of tumors including colon cancer [33]. miR-98-5p is also involved in the regulation of autophagy and predicted as target genes in the Bcl2 family [34, 35]. Previously, miR-455 is also recognized among a set of 6 miRNAs that are deregulated in a pancreatic ductal adenocarcinoma model of chemoresistant and a mesenchymal phenotype [36] as well in esophageal carcinoma cell lines [37] whereas the same is also reported to be up-regulated in CRC [38], which is similar with our findings, up-regulated miR 455 was down-regulated after the radiation and SN38 treatment. Contrary result, i.e. significant down-regulation of miR-455 is also reported in colon cancer along with its possible inhibitory role in proliferation and invasion of CRC by targeting RAF proto-oncogene serine/threonine-protein kinase [39]. miR-155-5p and miR-146-5p were first identified as NF-κB regulatory key factors to innate and adaptive immune responses [40, 41]. It is also involved in *IL8* regulation like miR-155-5p by lowering SHIP1 expression [42], miR-155-5p antagonists also exhibit possible therapeutic value in chronic inflammatory diseases, by modulating activation of macrophages and the number of circulating granulocytic cells [43]. miR-155-5p is also reported to be up-regulated in colon cancer. A combination of miR-155 level in colon cancer with the serum CEA level (both pre- and postoperatively) can afford more accurate information for diagnosis and prognosis, especially for predicting recurrence and metastasis postoperatively [44]. Deregulation of miR-1 is also reported earlier in CRC [45]. The expression level of miR-127-5p is used for determining the likelihood of CRC recurrence [46]. miR-142-5p is un regulated after NGX6 transfection in colon cancer cells [47]. miR-202-5p is also frequently down-regulated in gastric cancer. Transcriptional factor Gli1 is a target of miR-202-3p and plays an essential role as a mediator of the biological effects of miR-202-5p in gastric cancer. MiR-202-5p also inhibits the expression of γ-catenin and BCL-2. It seems that miR-202-5p may function as a novel tumor suppressor in gastric cancer, and its anti-tumor activity may attribute the direct targeting and inhibition of Gli1 [48].

The chronic inflammatory microenvironment consists of immune, inflammatory and stromal cells, all of which produce cytokines, growth factors and adhesion molecules that may sustain tumor growth, progression and spreading [49, 50]. In the present study, cytokines, IL-6, TNF-α, IL-1β, Il-4, IL-10, VEGF, and chemokines, IL-8, MIP-1α increased, and IFN-γ expression decreased after radiation, whereas, IL-6, IFN-γ, TNF-α, IL-1β, Il-4, IL-10 and IL-8 decreased, and VEGF and MIP-1α increased after SN38 treatment. Furthermore we found that the increased or decreased expression level of cytokines IL-6, TNF-α, IFN-γ, IL-1β, Il-4, IL-10, VEGF and of chemokines, IL-8, MIP-1α after radiation and SN38 treatment in a p53 dependent or independent manner.

Reduced IL-6 expression was observed after the SN38 treatment in all the cell lines regardless of p53 status. Commonly IL-6 involved in inflammation, cell growth, apoptosis, aging, and also induces an acute phase response. So decrease in IL6 expression possibly has some role in therapeutic aspect of colon cancer. A similar trend of reduction was noticed in case of IFN-γ, IL-1β, IL-4, which are considered pro-inflammatory in nature. It is reported that tumor associated macrophages protect colon cancer cells from TRAIL-induced apoptosis through IL-1β-dependent stabilization of Snail in tumor cells [51]. IL-1β also reported to promote epithelial-mesenchymal transition, stemness and invasiveness of colon cancer cells through Zeb factors [52]. Epithelial interleukin-4 receptor expression promotes colon tumor growth and mediates drug resistance in colon cancer stem cells [53, 54]. Increased IL-6 expression has been related to advanced stage and decreased survival in CRC patients [55]. Overexpression of IL-8 promotes tumor growth, angiogenesis, metastasis and chemoresistance, implying IL-8 to be an important therapeutic target in CRCs [56]. Utility of IL-8 as a biomarker for CRC detection and identification of high risk patients is also established [57]. Increased level of IL-10 is strongly associated with the progression of CRCs [58]. Our results also showed the reduction of IL-10 level after the SN38 treatment. IL-6 induced a STAT3-mediated IL-10 production in colon tumor cells is also reported [59]. Counter results - inverse association with tumor progression is also known [60]. In a nutshell, the immunostimulatory versus immunosuppressive effects of IL-10 marks it as a possible new therapeutic in tumor immunity [61]. IFN-γ (IFNG) is a pro-inflammatory cytokine that modulates many immune-related genes and shapes the tumor microenvironment in such a way that it shows both anti- and pro-tumorigenic activities [62–64]. VEGF-1 expression in CRCs is associated with disease localization, stage, identification of patients at high risk for disease progression and long-term disease-specific survival [65]. TNF-α promote colorectal tumorigenesis by stimulating glycolysis and growth factor production in CRC cells [66]. Interrupting tumor cell-macrophage communication by targeting TNF-α may provide an alternative therapeutic approach for the treatment of colon cancer [67].

We surprisingly found that IFN-γ did not interact with any of the 8 most up- and down-regulated miRNAs. Cytokines under consideration in general do not discriminate between up- and down-regulated miRNAs; interacts with both up- and down-regulated miRNA, except IL-4 and TNF-alpha, which interact only with up- and down-regulated miRNAs, respectively. let-7f-5p demonstrates the highest number of cytokine interaction, all other miRNAs, except miR-142-3p, exhibit interactions with multiple cytokines. It is possible that the production of various cytokines and their interaction with miRNA could play an important role in tumor growth and resistance or response to different treatment modalities. Hence, targeting tumor-producing cytokines and miRNAs could reduce tumor repopulation by eliminating tumor cells. Generally, the regulation of cytokine expression by miRNAs can be mediated through either direct binding of a miRNA to a target mRNA 3′UTR seed region in the cytokine mRNA, or by indirectly modulating adenine and uridine-rich elements binding proteins [68]. The interaction of miRNAs and cytokines contributes to the complex inter-cellular regulatory network, and might be a link between chronic inflammation and a variety of diseases, including colon cancer. The cytokines and growth factors produced by cancer cells function to create optimal growth conditions within the tumor microenvironment, while cytokines secreted by stromal cells may influence the behavior of malignant cells [69]. Therefore, it is necessary to develop a new approach for disrupting tumor-cytokine-miRNA networks to increase the treatment efficacy. Targeting miRNAs, therefore, may be a new therapeutic strategy for the treatment of colon cancer in the coming decades.

In the present study, we examined the expression of miRNAs, cyto- and chemokines, and hopefully the findings expanded our knowledge of p53 responses in colon cancer after radiation and SN38 treatment. However this study has several limitations for a deep understanding the biology of how these miRNAs affect CRC treatments and the pathways. Up- or down-regulated miRNA need to be validated through q-PCR. Their functional roles in more cell lines and even animal models should be studied. We also need to validate the functional roles of these miRNAs and pro- or anti-inflammatory mediators to confirm its functional use in patients, especially in the patients with clinical trials of radiotherapy and chemotherapy. Ultimately, understanding how changes in miRNA expression and function at each of these miRNA contribute will be necessary to fully understand the p53 responses in CRC pathogenesis. The accumulated data of dysregulated miRNAs may be incorporated into diagnosis and therapies of CRC patients and used for better treatment selection. If we could consider to use anti- or mimics of miRNAs or control the level of cyto- or chemokines, it might be more effective way of treatment of colon cancer rather than alone conventional treatments. The outcome of this investigation help to identify the most important miRNAs and cyto- or chemokines related to radiation and SN38 treatment in colon cancer to be targeted, which may have significant clinical importance for better management of colon cancer in the future.

In summary, after radiation and SN38 treatment, we found that most significant up- or down-regulation and interactions of 8 miRNAs (let-7f-5p, miR-455-3p, miR-98, miR-155-5p, miR-1, miR-127-5p, miR-142-5p, miR-202-5p), 7 cytokines (IL-1β, IL-4, IL-6, IL-10, IFN- γ, VEGF, TNF- α) and 2 chemokines (IL-8, MIP-1-α) were dependent on p53 status in colon cancer cells. Still now, the successful development of miRNA-based therapeutics for CRC has many challenging hurdles to overcome. The best miRNAs to be targeted in CRC are yet to be defined.

## MATERIALS AND METHODS

### Cell lines and cell culture conditions

Three human colon carcinoma cell lines, HCT116, were a kind gift from Dr. Vogelstein (Johns Hopkins University). HCT116, which produces wild-type p53 (HCT116^p53+/+^), heterozygous p53 (HCT116^p53+/−^), which has a single p53 allele disrupted and its p53-null counterpart HCT116^p53−/−^, in which the both alleles of p53 were deleted by means of homologous recombination [70]. The HCT116 cells with truncated p53 and missing 40 amino acid residues are considered functionally p53 negative [70]. The cells were maintained in McCoy's 5A medium (Sigma-Aldrich, St. Louis, MO) supplemented with 10% FBS (GIBCO, Invitrogen, Carlsbad, CA), 1.5 mM L-glutamine (GIBCO) and 1X PEST (GIBCO) at 37°C in a 5% CO_2_ incubator.

Other 2 human colon carcinoma cell lines, KM12C and KM12L4a (p53 mutant) with different metastatic potential, were kindly provided by Prof. Isaiah Fidler (Anderson Cancer Center, Houston, TX). The cell lines were maintained in Eagle's MEM medium supplemented with 10% heat-inactivated fetal bovine serum, sodium pyruvate, vitamins and a cocktail of penicillin and streptomycin at 37°C in 5% carbon dioxide. Cells growing in the exponential phase were harvested at approximately 80% confluency and used for the experiments.

### Radiation procedure

To evaluate the effect of radiation, the cells were seeded at a density of 1 × 10^5^ cells in 9.5 cm^2^ surface area plates, and radiated with a 6 MV photon spectra using a linear accelerator (Clinac 4/100, Varian; PaloAlo, CA). The cells were positioned below 3 cm PMMA, 105 cm from the photon source (the distance from the photon source to the PMMA-surface was 100 cm). Acrylic glass plates were placed above (3 cm thick) and underneath (10 cm thick) the cells. The cells were exposed to 2Gy radiation at room temperature. Following radiation on the three HCT116 cell lines, the cells were harvested after 2 hr for analysis of miRNAs and 2 hr, 48 hr for cytokines and chemokines analysis. For the both cytokine and chemokine analyses, the cell supernatants were used.

### SN38 treatment of colon cancer cells *in vitro*

The cells were plated at a density 50000 cells/cm^2^. Freshly prepared SN38 (Tocris Bioscience, UK) was added 24 hr after seeding the cells. The cells were further cultured for 48 hr before harvesting for miRNA extraction, and three HCT116 cell supernatant were used for the cytokines and chemokines analysis. Cancer cells with 50% growth inhibition after SN38 treatment were considered IC_50_ dose of SN38 and were used for the present experiment.

### Assay for determination of IC_50_ for SN38

Cellular viability and IC_50_ for SN38 were determined using the Cell Proliferation Kit II (XTT) according to the manufacturer's instructions (Roche Applied Science, Indianapolis, IN). The cells were seeded at a density of 5000 cells/cm^2^ in 96 well plates and allowed to attach for 24 hr. Freshly prepared SN38 (Tocris Bioscience, UK) was then added with a final concentration ranging from 0 to 1000 nM. After 48 hr of treatment cellular viability was determined using the XTT assay. The assay is based on the cleavage of the yellow tetrazolium salt XTT (2, 3-Bis (2-methoxy-4-nitro-5-sulfophenyl)-2H-tetrazolium-5-carbox-anilide) into a soluble orange formazan dye. This reaction is attributed mainly to the succinate-tetrazolium reductase system in the mitochondria of metabolically active cells. The absorbance measured at 450 nm is proportional to the number of viable cells. Experiments were repeated two times independently for cytokine and chemokine analysis.

### miRNA extraction

After 2 hr of 2Gy radiation treatment as well as after 48 hr of SN38 treatment, total RNA including was extracted using the *mir*Vana™ miRNA Isolation Kit (Invitrogen, Catalog Number AM1560).

### Reverse transcription and miRNA qPCR arrays

Expression profiling of miRNAs was performed by using TLDAs. TaqMan® Array Human MicroRNA Card A Set v2.0 (Applied Biosystems, Foster City, CA) enabled quantification of 384 human miRNAs and U6 snRNA (001973) endogenous controls that was used for data normalization. Megaplex miRNA RT primers with special stem-loop structure allowed synthesis of all cDNAs. A reverse transcription reaction was performed by using the TaqMan® MicroRNA reverse transcription kit and Megaplex™ RT primers, human pool A v2.0 (Applied Biosystems, Foster City, CA). The cDNA samples were pre-amplified by using Megaplex™ Pre Amp primers and TaqMan® Pre amp master mix (Applied Biosystems).

The reverse transcriptase mixture consisted of 100 ng of RNA sample, 100 mM of dNTPs, 50 U/ml of MultiScribe reverse transcriptase, 0.80 ml/reaction Megaplex RT primers (10X), 0.80 ml/reaction10X RT buffer, 20 U/ml RNase inhibitor and 25 mM MgCl2 (all part of TaqMan MicroRNA Reverse Transcription kit; Applied Biosystems). Reaction mixtures were incubated for 2 min at 16°C, 1 min. at 42°C and 1 sec. at 50°C for 40 cycles, then 5 min. at 85°C and finally held at 4°C (Gradient thermal cycler; Applied Biosystems). Pre-amplification reaction was also performed according to manufacturer's instruction for 12 cycles. Real-time PCR was performed using the Applied Biosystems 7900 HT Version 2.3 Sequence Detection System. Each 900 ml PCR reaction mixture consisted of 9 ml of diluted pre amplified RT product, 450 ml of TaqMan (No UmpErase UNG) Universal PCR Master Mix and 441 ml of nuclease free water. Then 100 ml of each PCR reaction mix was dispensed into each port of the TaqMan MicroRNA Array. Reactions were run at 50°C for 2 min., 95°C for 10 min., followed by 40 cycles at 95°C for 30 sec. and 60°C for 1 min. The expression levels of 379 miRNAs and 5 control assays were analyzed using the TaqMan® MicroRNA Array Set v2.0; Card A (Applied Biosystems).

### Data analysis

Ct (Cycle threshold) values were calculated using the SDS software (Applied Biosystems). Using the statistical computing environment R raw Ct values were then normalized with qPCRNorm quantile normalization, a data-driven normalization strategy for high-throughput qPCR data [71]. The comparative threshold cycle method was used to calculate the amplification factor. Radiation or drug-induced modulation of miRNA gene expression was calculated by the ΔΔC_t_ method, using the untreated cells as calibrator sample. *N*-fold change of the miRNA expression between treated and untreated samples was obtained using the formula 2^−ΔΔCt^. The data is presented as log_2_ (T/N) values, where log_2_ (T/N) was calculated as $log_{2}(T/N)=log_{2}\frac{2_{rumor}^{-\Delta \Delta \text{Ct}}}{2_{Normal}^{-\Delta \Delta \text{Ct}}}$. Values above 0.5 were considered as up-regulation of miRNAs. Heatmap provides a convenient way to visualize clustering of features and samples at the same time, and show the levels of values. Heatmap can be based on either Pearson correlation coefficients or Euclidean distance clustering. Euclidean-based heatmap will focus on the magnitude of different values, whereas Pearson clusters the samples based on similarities between the different profiles. Here Euclidean-based heatmap is used to show the levels of log_2_ (T/N) values from different therapy data. Further statistical analysis and visualization were performed by HTqPCR, a package of R that enables the processing and analysis of high-throughput quantitative real-time PCR data [72].

### Data normalization and EBarrays statistical analysis

The Empirical Bayes mixture method implemented in the R-package EBarrays [73] was used for analyzing differential expression (DE) of miRNAs in the colon cancer cells. Initially we normalized the Ct values (*Ct_norm_*). The *Ct_norm_* values were then transformed to an expression value $E_{ij}=-\left(Ct_{norm_{ij}}-40\right)$, where “*i*” is the index for each miRNA and “*j*” the index for treatment (*j* = 1 for untreated and *j* = 2 for treatment). After that we calculated the differential expression for each miRNA as $DE_{i}=E_{i_{1}}-E_{i_{2}}$ (positive values upregulated and negative values downregulated). The moderated *t*-statistics for each $DE_{i}$ were obtained and plugged into the local false discovery rate (threshold on the false discovery rate to be 0.01) procedure to find the significant *DE_i_* adjusted for multiple comparisons. The numbers of overlapping significant miRNAs between the cell lines were visualized with Venn-diagrams.

### Cytokine and chemokine analysis in Meso Scale Discovery instrument

HCT116^p53+/+^, HCT116^p53+/−^ and HCT116^p53−/−^ cell lines were treated by 2Gy radiation and SN38, respectively, and cell supernatant were collected. Following treatment the samples were centrifuged and supernatant was collected for further study in a cytokine and chemokine assay by Meso Scale Discovery (MSD). Cytokines (human cytokine 30 plex kit, V-plex, catalogue number K15054D-1, MSD, MD) including GM-CSF, IL-15, IL-1α, IL-16, IL-5, IL-17, IL-7, TNF-β, IL-12/23p40, VEGF and pro-inflammatory cytokines IFN-γ, IL-8 (high-ab), IL-1β, IL-10, IL-2, IL-12p70, IL-4, IL-13, IL-6, TNF-α, and 10 chemokine (human cytokine 30 plex kit, V-plex, catalogue number K15054D-1, MSD, MD) including MIP-1α, MIP-1β, TARC, Eotaxin, Eotaxin-3, MCP-1, MCP-4, MDC, IP-10 and IL-8 (chem) were analyzed by the assay. The Human Cytokine 30-Plex Panel Kit provides assay-specific components for the quantitative determination of 30 cytokines in human cell culture supernatants. Measurements were taken in a SECTOR Imager 2400 instrument (Meso Scale Discovery, MD). The principle of MSD is based on a reaction in which an electron transfer in electrochemically generated intermediates causes these molecules to enter an excited state. Once, excited these molecules can emit a photon of light when re-entering a lower energy level. Initially, capture antibodies are coated onto the surface of a plate. Samples are then incubated on the plate followed by the addition of an electrochemiluminescent tagged antibody. Analysis of this plate reveals luminescent regions in which specific interactions have occurred between the antibodies and analyte, allowing both a quantitative and qualitative analysis of the desired compound. This method is highly sensitive, has low background, does not incorporate washing steps and most importantly, allows the detection of multiple analytes at the same time. For all of the samples and all of the analytes a z-score was calculated as $z=\frac{X-\mu }{\sigma }$. Samples were considered up-regulated if the *z*-score was greater than 1.96 and down-regulated if the *z*-score was less than −1.96, corresponding to a *p*-value of 0.05.

### Pathway analysis of miRNAs

The regulatory effect of miRNAs in biological processes is normally executed by groups of these non-coding RNAs that act in a coordinated manner. The clustering and grouping of the target genes of miRNAs were performed to investigate the overall effect of the miRNAs regulatory effect. DIANA-miRPath [74] is an online computational application that identifies potentially altered biological pathways by the expression of single or multiple miRNAs, which is based on miRNA and pathway related information obtained from miRBase [75] and KEGG [76]. DIANA-miRPath can predict miRNA target genes by high accuracy based on DIANA-microT-CDS [77] or by experimental verification from Tarbase and then perform different analysis to them, like hierarchical clustering. We chose 4 most significant up-regulation miRNAs and 4 most significant down-regulating miRNAs to predict their relationship with KEGG pathways by using DIANA-miRPath.

### Relationship of miRNAs, cyto- and chemokines

By using online tools including TargetScan [78], PicTar [79], miRanda [80], DIANA-microT [81], RNA22 v2 [82], RepTar [83], and PITA [84] with default parameters, we predicted the possible interactions of miRNAs, cyto- and chemokines. The analyses were based on most up-regulated and most down-regulated of the miRNAs as well as cyto- and chemokines that increased or decreased significantly, after the radiation and SN38 treatment depending on p53 status of all the three cell lines.

## SUPPLEMENTARY FIGURES


