# Supplementary material for: Radiation and SN38 treatments modulate the expression of microRNAs, cytokines and chemokines in colon cancer cells in a p53-directed manner
Source: Oncotarget. 2015 Nov 5;6(42):44758–80. doi: 10.18632/oncotarget.5815 (PMC4792590; doi:10.18632/oncotarget.5815)
Supplement: Supplementary file 1 [file oncotarget-06-44758-s001.pdf]

SUPPLEMENTARY FIGURES

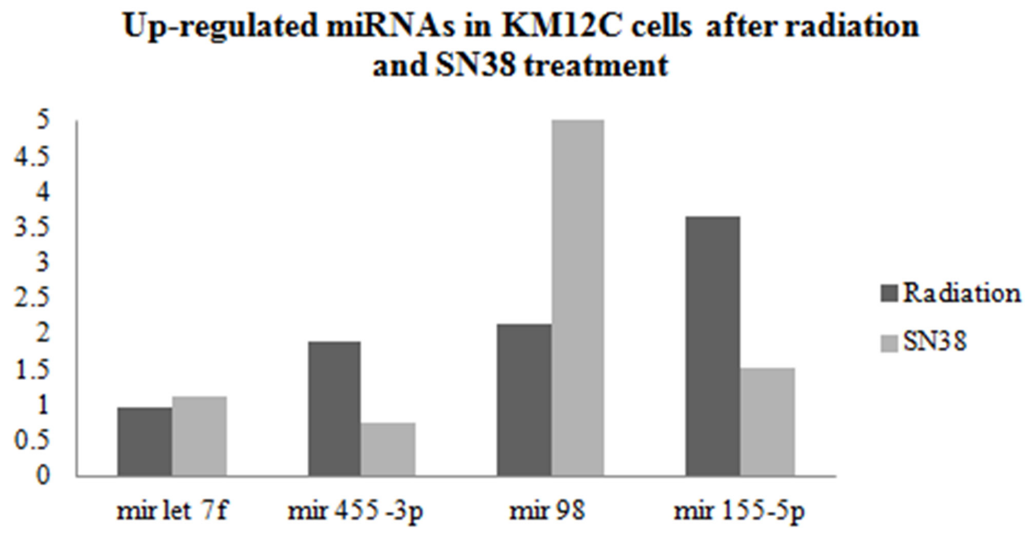

Supplementary Figure S1: The expression of let-7f-5p, miR-455-3p, miR-98, miR-155-5p miRNAs after radiation and SN38 treatment in human colon cancer cell lines: KM12C cells.

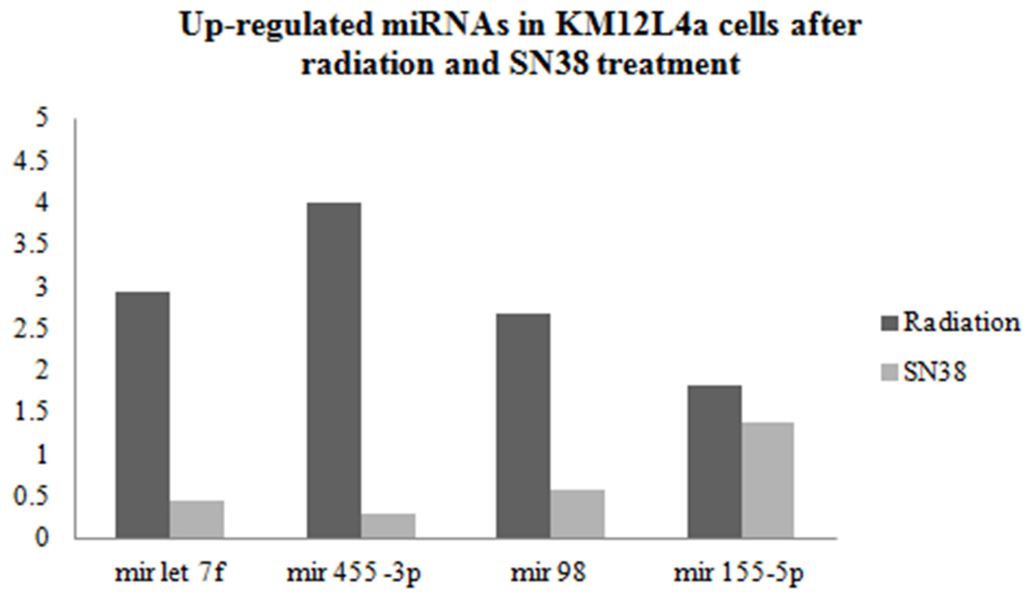

Supplementary Figure S2: The expression of let-7f-5p, miR-455-3p, miR-98, miR-155-5p miRNAs after radiation and SN38 treatment in human colon cancer cell lines: KM12L4a cells.
